# Quality of Life in Women with Endometriosis: The Importance of Socio-Demographic, Diagnostic-Therapeutic, and Psychological Factors

**DOI:** 10.3390/jcm14124268

**Published:** 2025-06-16

**Authors:** Agnieszka Bień, Aleksandra Pokropska, Joanna Grzesik-Gąsior, Magdalena Korżyńska-Piętas, Agnieszka Pieczykolan, Marta Zarajczyk, Roya Ali Pour, Adrianna Frydrysiak-Brzozowska, Ewa Rzońca

**Affiliations:** 1 Chair of Obstetrics Development, Faculty of Health Sciences, Medical University of Lublin, 20-081 Lublin, Poland; agnieszka.bien@umlub.pl (A.B.); magdalena.korzynska-pietas@umlub.pl (M.K.-P.); marta.zarajczyk@umlub.pl (M.Z.); 2 GEMELLI Private Health Care Center of Gynecology and Obstetrics, 31-559 Cracow, Poland; aleksandra.pokropska@gmail.com; 3 State University of Applied Sciences in Krosno, 38-400 Krosno, Poland; joanna.grzesik-gasior@pans.krosno.pl; 4 Independent Researcher, 20-562 Lublin, Poland; roya.alipour1363@gmail.com; 5 Institute of Nursing, Department of Integrated Medical Care, Faculty of Health Sciences, Collegium Medicum, The Masovian University in Płock, 09-402 Płock, Poland; a.frydrysiak-brzozowska@mazowiecka.edu.pl; 6 Global Newborn Society, Clarksville, MD 21029, USA; 7 Department of Obstetrics and Gynecology Didactics, Faculty of Health Sciences, Medical University of Warsaw, 00-575 Warsaw, Poland; ewa.rzonca@wum.edu.pl

**Keywords:** endometriosis, quality of life, self-efficacy, optimism, women’s health, psychosocial predictors, reproductive health

## Abstract

**Background**: Endometriosis is a chronic, estrogen-dependent inflammatory condition, that not only leads to significant physical symptoms but also exerts a profound psychological and social burden. This study aimed to asjsess the relationship between quality of life (QoL) in women with endometriosis and selected socio-demographic, diagnostic-therapeutic, and psychological factors, emphasizing self-efficacy and dispositional optimism as potential protective resources. **Methods**: A cross-sectional survey was conducted between 2020 and 2022 in healthcare facilities in eastern Poland. The study included 425 women diagnosed with endometriosis. The research tools were the Endometriosis Health Profile, the General Self-Efficacy Scale, and the Life Orientation Test-Revised, as well as an original socio-demographic and clinical questionnaire. Data were analyzed using descriptive statistics, linear regression, and hierarchical regression to assess the predictive role of psychological resources beyond sociodemographic and clinical variables. **Results**: A higher number of physicians from various specialties consulted before diagnosis was significantly associated with lower QoL in all EHP-30 domains except infertility (*p* < 0.05). The perceived economic burden of treatment was significantly related to lower QoL across all domains (*p* < 0.05). In contrast, higher levels of self-efficacy and dispositional optimism emerged as independent protective factors, positively associated with emotional well-being, social support, sexual functioning, and relationships with medical staff (*p* < 0.05). Psychological variables accounted for an additional 8.1% of the variance in QoL beyond socio-demographic and clinical predictors. **Conclusions**: The findings support the relevance of a biopsychosocial framework in managing endometriosis. Psychological resources play a critical role in coping with the disease and should be integrated into personalized care strategies.

## 1. Introduction

Endometriosis is a chronic, oestrogen-dependent inflammatory disease that affects approximately 190 million women and people assigned female at birth worldwide [1]. This condition is characterised by endometrium in locations other than the uterine cavity—most commonly within the pelvis, on the ovaries, fallopian tubes, peritoneum or rectovaginal septum. Ectopic foci of endometrium undergo hormonal cycling, leading to an inflammatory response, fibrosis, adhesion development, and cysts [2,3]. Clinical symptoms mainly include chronic pelvic pain, painful menstruation (dysmenorrhoea), pain during intercourse (dyspareunia), as well as complaints of urination or bowel movements and infertility. The disease can significantly affect women’s daily functioning, in terms of their work activities, family life, and personal relationships [1].

The literature increasingly emphasizes the psychological and social consequences of endometriosis, which can be as severe as the somatic symptoms. The chronicity of symptoms, their unpredictability, and impact on various areas of life contribute to the occurrence of emotional disorders, such as anxiety, irritability, a sense of helplessness, and depression [4,5].

Additional burdens include difficulties in social and professional life—many patients experience a lack of understanding from the community, insufficient support or downplaying of symptoms by medical staff, which can lead to isolation and stigmatisation. Women with endometriosis are more likely to experience deterioration in interpersonal relationships, particularly in the context of intimate and partnership life, as well as reduced self-esteem due to health difficulties, including fertility problems [6,7].

In this context, the need for a holistic approach to treatment that includes both the somatic aspects and the psychosocial functioning of patients is increasingly postulated. The identification of psychological resources that may have a protective function against illness-related stress is an important area of research into the quality of life (QoL) of women with chronic illnesses. Among these psychological resources, self-efficacy and dispositional optimism are particularly notable. Self-efficacy refers to one’s belief in their capacity to manage life challenges. Dispositional optimism is a general attitude towards the future—higher levels of optimism are associated with better emotional functioning and greater mental resilience under long-term health stress [8,9].

Studies show that individuals who are characterised by high self-efficacy and positive attitudes towards the future are less likely to experience depressive and anxiety symptoms and have higher levels of life satisfaction. In the context of a chronic condition like endometriosis, the availability of these psychological resources may enhance symptom management and elevate perceived QoL [9,10]. Medical and psychosocial aspects must be considered for a more comprehensive understanding of the impact of endometriosis on women’s everyday functioning and identification of potential protective factors against the negative consequences of the disease. However, despite growing interest in psychological determinants of QoL, few studies have simultaneously examined the role of both self-efficacy and dispositional optimism in women with endometriosis. The combined analysis of these two constructs using validated instruments, while also accounting for sociodemographic variables, offers a novel contribution to the literature. This integrative approach aims to better capture the psychological resilience mechanisms that support women’s daily functioning in the context of a chronic illness. Unlike many previous studies, this research integrates both the core and modular parts of the EHP-30 questionnaire, offering a more nuanced view of how endometriosis impacts specific life domains, including work and patient–physician relationships.

The study aimed to analyse the relationship between QoL in women with endometriosis and selected sociodemographic and psychological factors, with a particular emphasis on the role of self-efficacy and dispositional optimism as potential protective resources supporting daily functioning in the context of a chronic disease. We hypothesized that self-efficacy and optimism would positively predict QoL across EHP-30 domains.

## 2. Materials and Methods

### 2.1. Study Design and Sample Size

The study was conducted between 2020 and 2022 in health care centres in the Lublin Province (Poland). Questionnaires were distributed to all women with a confirmed diagnosis of endometriosis (ICD-10 code, N80 with various extensions) who came to the clinic for complaints related to the disease or for follow-up appointments, such as PAP smear, were over 18 years of age, and spoke Polish. The study excluded participants who were under the age of 18, were postmenopausal, pregnant, or had other chronic diseases (e.g., cancer, severe mental illness, autoimmune disorders) that could impair the study’s results, as well as those who did not understand Polish. First-time patients were eliminated to ensure diagnostic certainty and to include only women with prior experience with the condition and its therapy. A total of 425 women with endometriosis took part in the study. The participants were recruited as part of a cross-sectional survey conducted in available health care facilities treating women with endometriosis in the region. Due to the limited availability of this specific population (women with a confirmed diagnosis of endometriosis coded as ICD-10 N80), a convenience sampling method was used. This means that all eligible women who were present at the facilities during the study period were invited to take part in the study. To ensure that our sample size was adequate for the planned statistical analyses, we conducted a post hoc power analysis using G*Power, version 3.1 (Heinrich-Heine-University Düsseldorf, Düsseldorf, Germany). Based on the observed effect size (R^2^ = 0.20, f^2^ = 0.25), a significance level of α = 0.05, and seven predictors in the regression model, the calculated statistical power was greater than 0.999. This is well above the commonly accepted threshold of 0.80, indicating that our sample size (*n* = 425) was sufficient for the analyses conducted.

### 2.2. Data Collection

The study used a diagnostic survey method, the technique was questionnaire survey, and the research instrument consisted of both standardised research tools: Endometriosis Health Profile (EHP), Life Orientation Test-Revised (LOT-R), and General Self-Efficacy Scale (GSES), as well as the author’s interview questionnaire, including questions on socio-demographic variables (age, place of residence, education, marital status, professional situation, economic conditions, having children) and diagnostic-therapeutic variables (number of doctors consulted before diagnosis, duration of treatment, impact of treatment on the financial situation of women with endometriosis).

The Endometriosis Health Profile (EHP) was developed by Jones et al. (2001) as a tool to measure the QoL of women with endometriosis [11]. It consists of two parts: a basic form and a modular form. The core part contains 30 items and applies to all female respondents. 

It includes five thematic scales relating to important areas of psychosocial functioning: pain (11 items), control and powerlessness (6), emotional well-being (6), social support (4), and self-image (3). The modular section is designed for women who are affected by an aspect of their lives and allows a more detailed assessment of the impact of the disease on functioning in six areas: work life (5 items), relationships with children (2), sexual intercourse (5), feelings about medical profession (4), feelings about treatment (3), and feelings about infertility (4). Scores for each scale are converted into a range from 0 to 100 points (the total score for each scale is divided by the possible number of points and multiplied by 100). A lower score indicates a better QoL in a given area. Cronbach’s α coefficients for the core part range from 0.83 to 0.93 and for the modular part from 0.79 to 0.96. The use of the tool was approved by Clinical Outcomes at Oxford University Innovation [11].

The General Self-Efficacy Scale (GSES) by Schwarzer and Jerusalem, adapted into Polish by Juczyński, is used to measure an individual’s general belief in his or her ability to successfully cope with difficult life situations. The scale consists of 10 statements rated on a four-point Likert scale (1–4). The possible total score ranges from 10–40 points and is converted into sten values (1–4—low, 7–10—high). The reliability index (Cronbach’s α) is 0.85 [12].

The Life Orientation Test-Revised (LOT-R), developed by Scheier, Carver, and Bridges and adapted into Polish by Poprawa and Juczyński, is used to measure dispositional optimism. The tool contains 10 items, of which six have a diagnostic function and the others are control items. Responses are given on a five-point scale (0–4), giving a possible final score of 0–24 points. A higher score indicates a higher level of optimism, and the values obtained are interpreted in three ranges: 17–24 (optimism), 13–16 (medium level), 0–12 (pessimism). The reliability of the scale (Cronbach’s α) is 0.76 [13].

The study was conducted by the principles of the Declaration of Helsinki, and its implementation received a positive opinion from the Bioethics Committee at the Medical University of Lublin (no. KE-0254/256/2020, date 26 November 2020). All respondents were informed that participation in the study was voluntary and anonymous, that they could withdraw from the study at any time, and that the data collected would be used only for scientific purposes.

### 2.3. Statistical Analysis

Data were compiled using IBM SPSS Statistics, version 29 (IBM Corp., Armonk, NY, USA). Quantitative variables were described by mean, standard deviation, median, minimum and maximum value. The distribution of quantitative variables was assessed using the Shapiro–Wilk test, while homogeneity of variance was checked using the Levene’s test. Linear regression analysis was used to analyse the relationships between variables and included socio-demographic, diagnostic-therapeutic and psychological variables. Regression models were built separately for the overall QoL score (EHP-30) and each of the subscales of this tool.

A hierarchical regression analysis was conducted to determine the strength and significance of each group of variables. In the first step (Model 1), socio-demographic and diagnostic-therapeutic variables were included, while in the second step (Model 2), psychological variables (GSES and LOT-R) were included. By comparing the models, the increment in explained variance of QoL (ΔR^2^) resulting from the inclusion of psychological resources was determined. Test results with a *p*-value < 0.05 were considered statistically significant.

## 3. Results

### 3.1. Characteristics of the Study Group

Table 1 shows the characteristics of the women who participated in the study. The mean age of the respondents was 31.07 years (SD = 6.45). Most of the respondents lived in a voivodeship city (36.2%), were married or in a civil partnership (58.1%), had a university degree (68.2%), and were professionally active (72.7%). The majority of women declared that they had satisfactory socio-economic conditions (85.6%) and that they had no children (59.1%). The mean number of doctors the women consulted before the diagnosis of endometriosis was 4.60 (SD = 4.41). The mean duration of treatment for endometriosis was 3.41 years (SD = 4.39). More than three-fifths of the women (71.5%) felt that the expenses associated with the treatment of the disease affected their economic situation.

### 3.2. Mean Scores of Quality of Life, Self-Efficacy, and Optimism

Table 2 shows the mean scores for overall QoL and its components in the group of women with endometriosis. The mean score for overall QoL was 30.21 (SD = 26.65). The highest severity of difficulties was observed in the areas of infertility (M = 55.46; SD = 34.67) and treatment (M = 51.32; SD = 30.51). The mean self-efficacy score (GSES) was 27.89 (SD = 5.53) and the level of dispositional optimism (LOT-R) was 12.50 (SD = 4.73).

### 3.3. Regression Analysis

Table 3 shows the results of regression analysis between socio-demographic, diagnostic-therapeutic, and psychological variables of women with endometriosis. Lower QoL, as measured by the total score on the EHP-30 scale, was significantly associated with a higher number of doctors visited before the diagnosis of endometriosis (*p* < 0.001) and with the belief that treatment of the disease negatively affects one’s economic situation (*p* < 0.001). In contrast, higher self-efficacy and greater dispositional optimism were associated with better overall QoL (*p* < 0.001 for both). Across individual EHP-30 subscales, including pain, control and powerlessness, emotions, social support, and self-image, similar patterns were observed. More frequent consultations and negative economic perceptions were consistently associated with poorer QoL outcomes (*p* < 0.01), while greater self-efficacy and dispositional optimism were protective (*p* < 0.05), with optimism showing a particularly consistent effect. Additionally, in the self-image domain, older age emerged as a significant positive predictor of QoL (*p* = 0.043). Among the significant predictors, the number of doctors consulted before diagnosis and the perception of economic burden showed medium to large standardized effects (*β* ≈ 0.25–0.30), indicating a meaningful impact on overall QoL. In contrast, self-efficacy and dispositional optimism were associated with small to moderate effects (*β* ≈ −0.13 to −0.18).

Table 4 presents regression analysis results for QoL across six life domains specific to endometriosis: work life, sexual intercourse, infertility, treatment, medical care, and medical profession. Across all domains, lower QoL was significantly associated with a greater number of doctors consulted before diagnosis and with the belief that treatment negatively affects one’s economic situation (*p* < 0.001). In contrast, higher self-efficacy and dispositional optimism consistently predicted better QoL outcomes, particularly in domains related to treatment, sexual functioning, and medical care (*p* < 0.05). In the infertility domain, lack of experience with motherhood was strongly associated with lower QoL (*p* < 0.001). Interestingly, women with higher education reported lower QoL in the treatment domain (*p* = 0.047), suggesting possible differences in expectations or health literacy. These results reinforce the protective role of psychological resources and the detrimental impact of diagnostic delays and perceived financial burden. In terms of effect size, the most influential predictors across domains were the number of doctors consulted and perceived economic burden (*β* ≈ 0.24–0.31), both associated with lower QoL. Self-efficacy and optimism showed smaller but consistent positive associations (*β* ≈ −0.10 to −0.19).

A hierarchical regression analysis assessed the impact of socio-demographic characteristics, diagnostic-therapeutic variables, and psychological resources on the overall QoL of women with endometriosis. Sociodemographic and diagnostic-therapeutic variables were included in the first stage of the model (Model 1). In the second stage (Model 2), psychological resources were additionally included in the model: self-efficacy (GSES) and dispositional optimism (LOT-R). The results showed that the introduction of psychological variables in the second stage significantly increased the explained variance of QoL (ΔR^2^ = 0.081), indicating their significant independent contribution to explaining variation in QoL among women with endometriosis. Detailed results are presented in Table 5.

## 4. Discussion

Endometriosis, as a clinically and psychosocially complex disease, affects not only women’s physical health but also significantly interferes with their daily functioning and emotional well-being [1,4]. Our study confirmed a reduced QoL in women with endometriosis, though slightly higher than reported among women from the USA and Sweden, where less favourable health and psycho-physical functioning outcomes were observed [14,15]. In contrast, Dutch women demonstrated a comparable level of average QoL, with moderate severity of difficulties resulting from the symptoms of the disease [16]. Similarly, the Spanish population also showed poor outcomes, particularly in terms of emotional functioning and pain intensity [17]. The observed differences in QoL between countries could result from both different cultural backgrounds, the structure of healthcare systems and the individual experiences of patients, including the availability of diagnosis, the effectiveness of treatment or the level of emotional support they receive. As pointed out by Pontoppidan et al. (2023), the subjective QoL of women with endometriosis is largely shaped by clinical factors, such as the intensity of pain or the chronic nature of the symptoms [15]. This may also partly explain the observed differences between studies conducted in different countries and cultures [15]. The comparison of the results of our present study with those of other analyses confirms the multidimensional nature of the stresses experienced by women with endometriosis. It indicates the need for a comprehensive therapeutic approach that takes into account not only the somatic aspects, but also the emotional and social functioning of the patients.

### 4.1. Age

Among the examined demographic variables, age emerged as a factor related to perceived QoL. Our study suggests that younger women with endometriosis tend to report lower QoL than older patients. This may be due to several factors. Older women often experience longer disease duration and treatment, which promotes the development of adaptive strategies and reorganisation of daily functioning. They may also possess greater psychological resources and life experience, allowing them to reinterpret health difficulties more positively and strengthen their self-efficacy [18,19]. In contrast, younger women may experience stronger pressure to fulfil social roles such as motherhood, career advancement, or building stable relationships—roles that can be challenged by chronic illness and thus lower QoL [20]. The correlation between age and QoL was also confirmed by Hernández et al. (2022), who observed that women following treatment for endometriosis reported improvements with age, not only in physical functioning but also in mental health and vitality [21].

Notably, in our study, age correlated significantly only with scores on the ‘Self-image’ subscale. This may reflect the specific sensitivity of this domain to demographic differences, such as life stage or personal experience. The absence of significant correlations in other domains suggests that age does not directly influence all aspects of QoL, but may instead shape how women perceive themselves and their capabilities in the context of illness. Schwab et al. (2022) and Moore et al. (2023) reported comparable findings, highlighting that age and disease perception affect self-image and daily functioning in endometriosis [19,20].

### 4.2. Education

The results of our study showed that women with higher education reported lower QoL in the treatment domain. This may seem counterintuitive, as higher education is generally linked to better access to information, greater health awareness, and more active engagement with medical care. However, this finding may reflect elevated expectations regarding treatment quality and communication with healthcare providers among highly educated women. As previous studies have shown, individuals with university degrees are more likely to expect involvement in medical decision-making and tend to be more critical of ineffective interventions or organizational shortcomings in the healthcare system [22].

Education can increase patients’ awareness of their rights and healthcare standards, but it may also lead to disappointment when expectations are not met, particularly in chronic conditions like endometriosis, which require ongoing, individualized, and multidisciplinary care [23]. Women with higher education levels more often reported dissatisfaction with treatment and interactions with medical staff, suggesting that perceived lower quality of care may stem from unmet expectations rather than actual system shortcomings [24]. This underscores the need for individualized approaches and partnership-based communication, especially with well-informed and engaged patients.

### 4.3. Having Children

Endometriosis significantly increases the risk of infertility and miscarriage, with 25–50% of infertile women diagnosed with endometriosis and 30–50% of women with endometriosis experiencing infertility [25]. Fertility issues are major stressors and are associated with reduced QoL [26]. Our findings show that women who had not experienced motherhood reported lower QoL, suggesting that motherhood may play a key role in identity formation and psychological well-being [27]. Infertility can lead to grief, social isolation, and difficulties in daily functioning, negatively affecting mental health, interpersonal relationships, and self-esteem [28]. These results align with studies by Della Corte et al. (2020) and Bączek et al. (2024), which reported significantly reduced QoL in women with endometriosis, particularly when infertility was present [28,29]. For many, motherhood represents more than a biological function—it is deeply connected to personal identity and social roles. Its absence may result in feelings of unfulfillment and diminished QoL [30]. This highlights the importance of providing social support to those who do not conform to conventional notions of motherhood, enabling them to feel valued and accomplished in other areas of their lives.

### 4.4. Number of Doctors

The diagnostic process in endometriosis is often prolonged and fragmented, typically requiring multiple consultations with physicians from various specialties before a diagnosis is reached. This delay stems from personal, interpersonal, healthcare system, and disease-specific factors [31]. Our findings indicate that a higher number of pre-diagnostic consultations is significantly linked to poorer QoL across multiple EHP-30 domains, including pain, emotional well-being, social support, self-image, and the sense of control. These results align with previous studies highlighting the psychological burden of repeated consultations and diagnostic ambiguity in endometriosis [15,32]. More physician consultations before diagnosis were also associated with lower QoL in the professional life domain, reflecting the broader occupational impact of diagnostic delays on job stability, performance, and satisfaction. Similar results by Armour et al. (2020) and Melgaard et al. (2025) showed extended diagnostic paths lead to reduced economic independence, increased absenteeism, and diminished professional fulfilment [33,34].

In the treatment module, the link between multiple consultations and poorer QoL likely reflects cumulative frustration from delayed, ineffective, or inconsistent interventions. Agarwal et al. (2021) noted that women with longer pre-diagnostic histories were more likely to express scepticism toward treatments and showed lower adherence to therapies [35]. Although weaker, the relationship remained statistically significant in the sexual functioning module, with better outcomes linked to fewer consultations before diagnosis. Pontoppidan et al. (2023) similarly found that simpler care pathways improved satisfaction in intimate relationships and reduced psychological distress related to pain [15]. Our findings revealed a strong association between the number of physicians consulted prior to diagnosis and reduced perceived quality of patient–provider relationships. This suggests growing mistrust and emotional disengagement among patients navigating uncertain diagnoses. Mikesell and Bontempo (2023) emphasized that trust is a core dimension of patient- provider relationships in endometriosis and often compromised through fragmented experiences [36]. Hearn et al. (2024) observed that poor communication and repeated diagnostic failures fuel frustration, lower satisfaction, and decrease treatment adherence [37]. These relational disruptions not only affect emotional well-being but may weaken long-term healthcare engagement [37]. Large-scale epidemiological data by Melgaard et al. (2023; 2025) support this view: Danish case-control cohort studies found that women later diagnosed with endometriosis had significantly more pre-diagnostic visits, often in unrelated specialties—evidence of systemic diagnostic inefficiency [34,38].

### 4.5. The Impact of Medical Costs on the Economic Situation

Chronic gynaecological conditions like endometriosis impose significant economic burdens, negatively affecting patients’ psychological, social, and occupational functioning, and reducing productivity [28,39,40]. Our findings show that lower QoL across all domains of the Endometriosis Health Profile is closely associated with patients’ perceptions of the financial strain caused by treatment. The total cost of endometriosis includes both direct expenses, such as medical visits, prescriptions, hygiene products, and supplements [40,41], and indirect costs, primarily related to work absence, decreased productivity, and social limitations [42].

As demonstrated by previous economic analyses, the total cost of endometriosis per patient can amount to several thousand euros annually, with the greatest burden stemming from lost income and the social consequences of the disease. The chronic nature of the condition necessitates continuous access to healthcare, which further exacerbates the financial strain experienced by women living with this illness [28,41]. It should be emphasised that the personal costs for women with endometriosis are not only economic, but also psychological. These women often experience increased anxiety, depression, and chronic stress, which can lead to social isolation, feelings of hopelessness, and a negative self-image [43,44,45].

### 4.6. Psychological Resources

The next step in this study was to assess the role of self-efficacy and dispositional optimism as potential protective resources supporting daily functioning within the context of endometriosis. In the analysed group of women with endometriosis, a moderate sense of self-efficacy and a tendency towards pessimism in future projection were found. Particularly low levels of dispositional optimism are confirmed by the results of the LOT-R scale, which is in line with the findings of Morán-Sánchez et al. (2021), who showed that women with endometriosis have significantly lower optimism results compared to healthy women [46]. These mean values may have important implications for psychological adjustment to living with a chronic disease such as endometriosis. Reduced levels of optimism may hinder positive reinterpretation of difficult experiences, while moderate self-efficacy suggests limited confidence in respondents’ ability to cope with daily health challenges. Both self-efficacy and dispositional optimism are widely recognised in the literature as important psychological resources that foster higher levels of QoL. Studies in various clinical populations, including oncology and gynaecology patients, have shown that higher values of both traits are associated with lower levels of anxiety and depression, as well as higher life satisfaction and better emotional functioning [47,48]. In the case of endometriosis, the course of which is often characterised by chronic pain, lack of symptom control and limited treatment efficacy, the presence of positive psychological resources may have a buffering function, reducing the impact of disease-related stress [49]. Wojcieszek et al. (2023) found that individuals with higher self-efficacy cope better with chronic pain and report fewer depressive symptoms, regardless of disease severity [9]. Including self-efficacy and dispositional optimism in assessments of women with endometriosis can help identify psychological coping mechanisms and areas needing support. The moderate levels of these traits in our sample suggest they can be strengthened through integrated care. To explore these findings further, we also analysed the relationships between levels of self-efficacy and QoL in specific domains of psychosocial functioning.

### 4.7. Self-Efficacy

Self-efficacy, defined as the belief in one’s ability to act effectively and achieve goals, plays an important role in the adaptation to health-challenging situations such as chronic disease [8]. In the context of endometriosis—a condition requiring long-term treatment, numerous interactions with the health care system and adaptation to chronic symptoms—this resource can act as a buffer against the deterioration of QoL [9].

The results of our study confirmed that higher levels of self-efficacy were associated with better overall QoL for women with endometriosis. This relationship was evident in most of the analysed dimensions of the EHP-30 questionnaire. Women who were characterised by a stronger belief in self-efficacy functioned better not only in emotional, but also in social, sexual and occupational spheres. These results are consistent with the findings of previous studies, which emphasised that psychological efficacy contributes to better adaptation to disease symptoms, lower stress levels and increased emotional resilience [50,51]. In our study, we also observed that a higher sense of efficacy was associated with higher QoL scores in areas such as pain, sense of control and powerlessness, self-assessment and relationships with medical staff. The last element in particular may be important—people convinced of their self-efficacy are more likely to formulate expectations and actively cooperate with the doctor, which may result in greater satisfaction with the treatment [52].

Infertility is one of the most serious psychological strains associated with endometriosis. In our study, lack of motherhood experience was significantly associated with lower QoL in the ‘fertility’ domain. These findings support previous findings that infertility generates severe emotional stress, a sense of loss of control, and a disruption of social roles [53]. In these situations, psychological resources, including a sense of efficacy, can play a protective role, reducing negative emotional consequences. Women who are more resourceful and confident in their ability to control their lives adapt better to the challenges of fertility and maintain a higher QoL, despite difficulties in implementing maternity plans [54].

The positive link between self-efficacy and QoL in occupational and sexual domains is noteworthy. Women with higher self-efficacy reported greater work engagement and satisfaction with their intimate lives, despite disease-related challenges. This is supported by Orr et al. (2023), who found that strengthening self-efficacy can improve coping with sexual pain and discomfort [55]. These findings highlight self-efficacy as a protective psychological resource. Integrating interventions that enhance self-efficacy into endometriosis care may significantly improve patients’ daily functioning and overall QoL.

### 4.8. Dispositional Optimism

Our results indicate that dispositional optimism is a significant predictor of QoL in women with endometriosis. Notably, the mean LOT-R score in our sample falls within the pessimistic-to-moderate range, suggesting that this psychological resource may be limited among the participants. Higher levels of optimism are associated with better emotional functioning, perceived social support, and self-esteem. Similar findings were reported by Magariños López et al. (2022), who observed significant associations between pain severity and symptoms of anxiety and depression in women with chronic pelvic pain—a key manifestation of endometriosis [56]. According to Kalfas et al. (2022), quality-of-life interventions should include psychological assessments, including resilience, coping strategies, and hope for clinical improvement [57]. The importance of temperament and character traits in modulating pain perception and general well-being in endometriosis was also emphasised by Facchin et al. (2016) [58]. Our study further supports the view that higher optimism levels are associated with stronger perceived social support, consistent with studies suggesting that optimism fosters stable, supportive relationships in chronic illness contexts [59,60,61]. Patients with higher levels of optimism also rated the treatment process and relationships with medical staff more positively. Boehm et al. (2018) noted that optimistic individuals engage more with treatment, follow recommendations better, and adopt healthier behaviours [62]. In women with endometriosis, this trait is linked with lower pain perception and less helplessness.

Chronic pelvic pain, which is typical of the disease, represents a major medical as well as psychological challenge. As shown by Gustin et al. (2016), patients with chronic pain may co-occur with characteristics of certain personality disorders, further complicating the adaptation and treatment process [63]. Likewise, Facchin et al. (2016) pointed to a psychological profile in women with painful endometriosis that includes pessimism and low coping efficacy [58]. Although optimism did not significantly affect fertility- or work-related QoL domains, it was a significant predictor of sexual well-being. This suggests that cognitive traits like optimism may be more relevant to relational functioning than to externally constrained existential challenges. This underscores the potential benefit of targeted psychological interventions aimed at strengthening optimism in this population.

Both a sense of efficacy and dispositional optimism are consistently described in the literature as important resources that support adaptation to health situations requiring long-term adjustment [8,46]. Our findings reinforce that these traits enhance emotional and social functioning and improve overall QoL, even in the context of persistent disease. Other studies have similarly shown that higher levels of these traits correlate with lower anxiety and depression, and improved psychosocial functioning [59,64]. When viewed as resources, optimism and efficacy may buffer chronic health stress, reducing perceived burden and improving well-being [48,65]. Our results support incorporating psychological interventions that strengthen such resources into integrated care models for endometriosis, alongside pharmacological treatment and education. This study advances current knowledge by combining psychosocial predictors with detailed domain-specific quality-of-life measures, offering a novel, integrative perspective on patient functioning in endometriosis.

### 4.9. Strengths and Weaknesses of the Study

This study took a comprehensive approach to assessing the QoL in women with endometriosis by examining both overall functioning and specific life domains. The use of the full EHP-30 questionnaire, including the less commonly applied modular sections, allowed for a broader analysis of areas such as work, sexual functioning, and interactions with medical staff. This enabled more precise identification of support needs. A key strength was the inclusion of psychological variables—self-efficacy and dispositional optimism—which are crucial in coping with chronic illness. Their integration opens the door to more tailored psychological interventions. Additionally, hierarchical regression analysis helped isolate the unique impact of psychological resources beyond sociodemographic and clinical factors. The study also complements our earlier research on the medical determinants of QoL in this population [66].

As with any study, certain limitations are also present here. The lack of data regarding the stage of disease limits the precision of the analysis of the relationship between clinical characteristics and QoL. Another limitation is the heterogeneity of participants’ ages, which may influence coping strategies and perceived QoL. The cross-sectional design of the study does not allow for a full understanding of how disease burden changes over time. Given the irregular course of endometriosis, it would be advisable to conduct longitudinal studies to capture better the dynamics of the disease’s impact on QoL. Although the study has certain limitations, it offers important insights into the experiences of women living with endometriosis, and emphasizes the relevance of incorporating a psychological viewpoint when evaluating their QoL. Additionally, as the study was conducted exclusively in the Lublin Province, the findings may not be fully generalisable to other regions or healthcare systems. This regional limitation should be considered when interpreting the results.

### 4.10. Practical Implications

The findings of this study have direct implications for enhancing the support and care of women at risk of, or living with, endometriosis. The observed associations between psychological resources, healthcare experiences, and QoL point to several practical recommendations. A more integrated approach to diagnosis and treatment—focused on reducing costs and improving access to specialist care—can significantly improve patients’ well-being. A key component of this strategy should be the inclusion of psychological support aimed at strengthening self-efficacy and dispositional optimism, both identified as protective factors. Incorporating these elements into care can help tailor interventions to individual needs, ultimately improving clinical outcomes and patients’ overall experience of care.

Clinicians can strengthen self-efficacy and optimism through targeted, evidence-based psychological interventions. Cognitive behavioural therapy (CBT), for instance, has demonstrated efficacy in improving psychological functioning and reducing pain-related disability in women with endometriosis—both in adolescents and adults—by reinforcing adaptive coping strategies and reframing maladaptive cognitive patterns [67].

This study also identified a strong correlation between the number of healthcare providers consulted before diagnosis and a decline in perceived quality of doctor–patient interactions. Addressing this issue requires clinicians to adopt empathic, patient-centred communication practices that validate patients’ experiences and promote shared decision-making. Enhancing such interactions can mitigate the psychological burden associated with diagnostic delays and fragmented care pathways.

Given the link between delayed diagnosis and reduced QoL, especially in occupational, sexual, and treatment-related domains, faster diagnostic pathways are essential. This includes improving referral systems, training primary care providers, and incorporating content on chronic gynaecological pain and diagnostic barriers into medical education. Public awareness campaigns can promote earlier symptom recognition, reduce stigma, and foster empathy. Health policies should also support affected women through flexible work options and access to medical leave. Due to the complex nature of endometriosis, multidisciplinary care involving gynaecologists, psychologists, physiotherapists, and pain specialists is crucial for delivering personalised, comprehensive treatment, and improving long-term outcomes. It is recommended that clinicians routinely assess patient-centred outcomes using validated instruments such as the Endometriosis Health Profile-30 (EHP-30). Systematic monitoring enables the development of personalised care strategies and ensures ongoing responsiveness to changes in patients’ quality-of-life needs.

## 5. Conclusions

The main socio-demographic and diagnostic/therapeutic factors negatively affecting the QoL of women with endometriosis were the higher number of doctors consulted before diagnosis and the subjectively perceived economic impact of treatment costs. These factors were significantly associated with lower QoL scores in all core domains of functioning, particularly pain, sense of control and powerlessness, emotional well-being, level of social support, and self-image.

Psychological resources, such as self-efficacy and dispositional optimism, were found to be significant protective factors, supporting higher ratings of emotional, social, and sexual spheres, and in relations with medical staff. Furthermore, the impact of these variables remained independent of other sociodemographic and clinical factors, highlighting their specific role in adaptation to the disease.

Based on the obtained results, it seems reasonable to include psychological interventions as an integral part of the comprehensive treatment of women with endometriosis. The inclusion of emotional and social aspects in the therapeutic process can significantly contribute to improving the QoL of patients and increasing the effectiveness of coping with the chronic nature of the disease.

## Figures and Tables

**Table 1:** Characteristics of the study group.

Socio-Demographic Data		N	%
Age	M (SD) Me; Min-Max	31.07 (6.45) 30.00; 18.00–53.00	
Place of residence	Voivodship city	154	36.2
	Other city	141	33.2
	Village	130	30.6
Marital status	Married/in a civil partnership	247	58.1
	Single	178	41.9
Education	Other than university degree	135	31.8
	University degree	290	68.2
Professional work	Yes	309	72.7
	No	116	27.3
Socio-economic conditions	Satisfactory	364	85.6
	Unsatisfactory	61	14.4
Having children	Yes	174	40.9
	No	251	59.1
Number of doctors consulted before diagnosis	M (SD) Me; Min-Max	4.60 (4.41) 3.00; 0.00–30.00	
Duration of endometriosis treatment (years)	M (SD) Me; Min-Max	3.41 (4.39) 2.00; 0.00–31.00	
Opinion of whether the cost of treating an illness has an economic impact	Yes	304	71.5
	No	121	28.5

M—mean; Me—median; SD—standard deviation.

**Table 2:** Mean scores of quality of life (EHP-30), self-efficacy (GSES) and dispositional optimism (LOT-R) in a group of women with endometriosis.

Variables	M	Me	SD	Min	Max
EHP-30	30.21	26.74	26.65	0.00	82.16
Pain	36.20	29.55	32.01	0.00	84.09
Control & powerlessness	32.55	29.17	31.80	0.00	91.67
Emotional well-being	35.37	25.00	24.76	0.00	91.67
Social support	17.88	6.25	18.89	0.00	75.00
Self-image	29.04	25.00	33.06	0.00	91.67
Work life	39.69	40.00	29.49	0.00	100.00
Children	29.85	25.00	30.43	0.00	100.00
Sexual intercourse	44.46	45.00	32.28	0.00	100.00
Medical profession	43.61	43.75	32.85	0.00	100.00
Treatment	51.32	50.00	30.51	0.00	100.00
Infertility	55.46	62.50	34.67	0.00	100.00
GSES	27.89	29.00	5.53	10.00	40.00
LOT-R	12.50	12.00	4.73	0.00	24.00

M—mean; Me—median; SD—standard deviation; EHP-30—Endometriosis Health Profile-30; GSES—General Self-Efficacy Scale; LOT-R—Life Orientation Test-Revised.

**Table 3:** Regression analysis of quality of life and socio-demographic, diagnostic-therapeutic, and psychological variables of respondents—baseline questionnaire EHP-30.

Predictors	EHP-30 Total Score F = 44.607; *p* < 0.001; R = 0.737; R^2^ = 0.543; Adjusted R^2^ = 0.531		Pain F = 10.132; *p* < 0.001; R = 0.477; R^2^ = 0.228; Adjusted R^2^ = 0.205		Control and Powerlessness F = 10.884; *p* < 0.001; R = 0.491; R^2^ = 0.241; Adjusted R^2^ = 0.219		Emotional Well-Being F = 10.942; *p* < 0.001; R = 0.492; R^2^ = 0.242; Adjusted R^2^ = 0.220		Social Support F = 8.637; *p* < 0.001; R = 0.448; R^2^ = 0.201; Adjusted R^2^ = 0.178		Self-Image F = 10.642; *p* < 0.001; R = 0.486; R^2^ = 0.237; Adjusted R^2^ = 0.214	
	β	*p*	β	*p*	β	*p*	β	*p*	β	*p*	β	***p***
Constant	nan	0.000	nan	0.000	nan	0.000	nan	0.000	nan	0.000	nan	0.000
Age	−0.047	0.308	−0.064	0.214	−0.082	0.112	−0.047	0.362	−0.099	0.059	−0.104	0.043
Place of residence	0.056	0.164	0.027	0.554	0.035	0.437	0.022	0.628	0.029	0.520	0.045	0.317
Marital status	0.009	0.827	0.033	0.488	0.002	0.971	0.045	0.341	0.021	0.667	0.025	0.600
Education	−0.021	0.616	−0.045	0.325	−0.057	0.211	−0.076	0.094	−0.077	0.100	−0.041	0.364
Professional work	−0.016	0.710	−0.047	0.323	−0.036	0.446	−0.013	0.786	0.010	0.843	−0.015	0.750
Socio-economic conditions	−0.030	0.469	−0.042	0.370	−0.026	0.578	−0.066	0.154	−0.030	0.526	−0.043	0.358
Having children	−0.029	0.478	−0.080	0.077	−0.064	0.151	−0.061	0.175	0.010	0.834	−0.005	0.907
Number of doctors consulted before diagnosis	0.297	0.000	0.200	0.000	0.252	0.000	0.259	0.000	0.285	0.000	0.281	0.000
Duration of endometriosis treatment (years)	−0.005	0.915	0.017	0.729	0.007	0.879	0.016	0.733	−0.003	0.952	0.014	0.773
Opinion of whether the cost of treating an illness has an economic impact	0.299	0.000	0.199	0.000	0.184	0.000	0.185	0.000	0.125	0.008	0.152	0.001
GSES	−0.175	0.000	−0.150	0.003	−0.126	0.012	−0.083	0.098	−0.101	0.051	−0.153	0.002
LOT-R	−0.177	0.000	−0.133	0.011	−0.147	0.004	−0.182	0.000	−0.159	0.003	−0.150	0.004

**Table 4:** Regression analysis of quality of life and socio-demographic, diagnostic-therapeutic, and psychological characteristics of respondents (modular questionnaire).

Predictors	Work Life F = 10.972; *p* < 0.001; R = 0.492; R^2^ = 0.242; Adjusted R^2^ = 0.220		Sexual Intercourse F = 6.732; *p* < 0.001; R = 0.405; R^2^ = 0.164; Adjusted R^2^ = 0.140		Infertility F = 8.069; *p* < 0.001; R = 0.436; R^2^ = 0.190; Adjusted R^2^ = 0.167		Treatment F = 12.533; *p* < 0.001; R = 0.517; R^2^ = 0.267; Adjusted R^2^ = 0.246		Medical Profession F = 12.464; *p* < 0.001; R = 0.516; R^2^ = 0.266; Adjusted R^2^ = 0.245	
	β	*p*	β	*p*	β	*p*	β	*p*	β	*p*
Constant	nan	0.001	nan	0.000	nan	0.000	nan	0.008	nan	0.010
Age	−0.019	0.711	−0.054	0.320	0.062	0.239	0.030	0.558	0.067	0.183
Place of residence	0.046	0.295	0.069	0.140	0.025	0.582	0.039	0.372	0.062	0.159
Marital status	−0.045	0.347	0.070	0.157	−0.038	0.436	−0.034	0.464	−0.062	0.182
Education	−0.008	0.865	0.014	0.767	0.084	0.073	0.089	0.047	0.007	0.883
Professional work	0.025	0.591	0.001	0.990	0.047	0.340	0.018	0.697	−0.004	0.924
Socio-economic conditions	0.002	0.974	−0.034	0.482	−0.005	0.923	0.031	0.500	0.043	0.343
Having children	0.044	0.325	0.038	0.420	−0.279	0.000	0.083	0.061	0.005	0.915
Number of doctors consulted before diagnosis	0.246	0.000	0.094	0.044	0.054	0.239	0.236	0.000	0.300	0.000
Duration of endometriosis treatment (years)	0.004	0.927	−0.036	0.464	0.005	0.913	−0.038	0.415	−0.067	0.151
Opinion of whether the cost of treating an illness has an economic impact	0.313	0.000	0.241	0.000	0.127	0.008	0.312	0.000	0.256	0.000
GSES	−0.115	0.022	−0.141	0.008	−0.168	0.001	−0.038	0.445	−0.103	0.037
LOT-R	−0.066	0.198	−0.120	0.027	−0.042	0.434	−0.193	0.000	−0.103	0.042

**Table 5:** Hierarchical regression analysis of quality of life in women with endometriosis—influence of socio-demographic, diagnostic-therapeutic, and psychological factors.

Model	R^2^	*p*
Model 1—socio-demographic and diagnostic/therapeutic factors	0.306	<0.001
Model 2—+GSES and LOT-R	0.386	<0.001
ΔR^2^ (Model 2–Model 1)	0.081	–

## Data Availability

The data that support the findings of this study are available from the corresponding author upon reasonable request.

## Abbreviations

The following abbreviations are used in this manuscript:

QoL	Quality of Life
EHP	Endometriosis Health Profile
LOT-R	Life Orientation Test-Revised
GSES	General Self-Efficacy Scale
